# Comparing the Efficacy and Safety of Anti-CGRP Monoclonal Antibodies Versus Topiramate for Migraine Prophylaxis: Six-Month, Real-World, Intention-to-Treat Retrospective Evidence from the GRASP Study Group

**DOI:** 10.3390/neurolint18040067

**Published:** 2026-04-01

**Authors:** Michail Vikelis, Dimitrios Rikos, Andreas A. Argyriou, Panagiotis Soldatos, Christos Tsironis, Emmanouil Giakoumakis, Georgia Xiromerisiou, Maria Chondrogianni, Aikaterini Foska, Maria Koutsokera, Konstantinos Notas, Eleni Mavraki, Emmanouil V. Dermitzakis

**Affiliations:** 1 Glyfada Headache Clinic, 16675 Glyfada, Greece; 2 Neurology Department, 404 Military Hospital, 41222 Larisa, Greece; rikosd@hotmail.com; 3 Headache Outpatient Clinic, Neurology Department, Agios Andreas General Hospital of Patras, 26335 Patras, Greece; andargyriou@yahoo.gr; 4 Kalamata Headache Clinic, 24133 Kalamata, Greece; soldatosp@gmail.com; 5 Headache Outpatient Clinic, Department of Neurology, University Hospital of Ioannina, 46100 Ioannina, Greece; ctsiron@gmail.com; 6 NeuroCrete Headache Clinic, 71303 Irakleio, Greece; giakoumakis@neurocrete.gr; 7 Private Institute of Preventive Neurology and Brain Health-Mindful Mind, 55133 Thessaloniki, Greece; georgiaxiromerisiou@gmail.com; 8 Second Department of Neurology, National and Kapodistrian University of Athens, School of Medicine, “Attikon” University Hospital, 12461 Athens, Greece; mariachondrogianni@hotmail.gr (M.C.); dkfoska@gmail.com (A.F.); 9 Headache Outpatient Clinic, Neurology Department, Thriasion General Hospital, 19300 Elefsina, Greece; mkoutsokera@yahoo.gr; 10 Headache Outpatient Clinic, Saint Luke’s Hospital, 55236 Thessaloniki, Greece; konstantinos_notas@hotmail.com; 11 Headache Outpatient Clinic, Department of Neurology, University General Hospital of Alexandroupolis, 68100 Alexandroupolis, Greece; emavr2009@hotmail.com; 12 Headache Outpatient Clinic, General Clinic, 54645 Thessaloniki, Greece; manolis.dermitzakis@gmail.com

**Keywords:** real-world, comparison, effectiveness, safety, anti-CGRP monoclonal antibodies, topiramate

## Abstract

**Objective**: This retrospective, intention-to-treat real-world study, designed by the Greek Research Alliance for the Study of headache and Pain (GRASP) sought to compare the effectiveness and safety of anti-CGRP monoclonal antibodies (anti-CGRP Mabs) to topiramate in preventing migraine. **Patients and methods**: Patients received either fremanezumab, erenumab, galcanezumab, eptinezumab, or topiramate for at least six months. Outcomes included reductions in monthly headache days (MHDs), ≥50% and ≥75% responder rates, monthly acute medication intake (MAI), MHDs with peak headache intensity ≥5 on VAS, migraine-related disability (MIDAS, HIT-6), quality of life (EQ-VAS), discontinuation rates and safety. **Results**: We included 409 migraine patients (median age 45.2 years), predominantly female (80%) and mostly with long-standing disease and high baseline burden. After six months, all treatments reduced MHDs. Mean MHDs decreased by −7.8 days with anti-CGRP Mabs versus −3.8 days with topiramate (*p* < 0.001). Higher ≥50% and ≥75% responder rates were observed across all anti-CGRP agents, compared to topiramate. Anti-CGRP Mabs also achieved greater reductions in moderate/severe MHDs, MAI, disability metrics, and superior QOL gains. Among the CGRP-targeted therapies, slight differences in effectiveness outcomes were present, though failing to demonstrate any specific superiority. Safety was favorable for anti-CGRP Mabs, whereas topiramate showed substantially higher adverse events and discontinuations. **Conclusions**: Anti-CGRP Mabs were more effective, produced greater reductions in disability and higher improvements quality-of-life metrics and were better tolerated than topiramate. Differences among individual anti-CGRP agents were modest and unlikely to represent a clinically meaningful superiority, supporting a class-wide benefit vs. topiramate in migraine prevention both in terms of effectiveness and safety.

## 1. Introduction

Migraine is a highly prevalent and disabling brain disorder, characterized by recurrent headache attacks, interictal hypersensitivity, and a substantial personal, social, and economic burden [1]. In routine clinical practice, preventive treatment is indicated for a large proportion of patients with high-frequency episodic migraine (HFEM) or chronic migraine (CM), even more when attacks are prolonged, insufficiently controlled by acute therapies, or associated with marked disability and reduced quality of life [2]. Despite the availability of multiple preventive options, long-term migraine prevention remains challenging, as treatment decisions must balance efficacy, tolerability, comorbidities, patient preferences, and the likelihood of sustained adherence [3].

Within this therapeutic landscape, topiramate has long represented the standard-of-care oral preventive, based on guideline-level evidence and widespread availability and clinical experience. It is commonly prescribed as a first-line option, given its proven efficacy in reducing migraine frequency and low cost [4,5]. However, its real-world effectiveness is often substantially limited by tolerability issues, high discontinuation rates, and poor to modest benefit in difficult-to-treat populations [6]. Outside the controlled conditions of clinical trials, slow titration requirements, dose-limiting adverse effects, and neurocognitive side effects frequently undermine treatment adherence and persistence [7]. Paresthesias, cognitive decline, mood changes, weight loss, and nephrolithiasis are well-recognized reasons for treatment discontinuation, while teratogenic risk and overall tolerability concerns further complicate its use in women of childbearing potential [8]. These limitations are especially relevant in secondary and tertiary headache centers and clinics, where difficult-to-treat patients with long disease duration, multiple comorbidities, medication-overuse headache (MOH), and repeated failures of prior preventive therapies are often treated. In such settings, discontinuation due to adverse events is not an insignificant outcome, but a key determinant of observed effectiveness, as early treatment cessation precludes the possibility of sustained clinical benefit [6].

Monoclonal antibodies targeting the calcitonin gene-related peptide (anti-CGRP Mabs) pathway have revolutionized the treatment landscape in migraine prophylaxis [9]. These biological agents offer a mechanism-based approach to migraine prevention, with simplified dosing regimens and generally favorable tolerability profiles, even in patients with multiple prior preventive failures, compared to repurposed medications [10,11]. The currently available anti-CGRP Mabs, including erenumab, which targets the CGRP receptor, and fremanezumab, galcanezumab, and eptinezumab, which target the ligand, have some differences in pharmacological properties and also in the route and frequency of administration [12]. Pivotal clinical trials have consistently demonstrated clinically meaningful reductions in migraine frequency and severity, coupled with improvements in patient-reported outcomes, rapid onset of effect, and comparatively low rates of treatment discontinuation [13].

Nevertheless, the extent to which these clinical findings translate into routine clinical practice remains influenced by several factors that are often underrepresented in randomized studies. These include treatment sequencing after multiple prior preventive failures, heterogeneity in baseline disease severity, comorbidity burden, adherence patterns, and national reimbursement policies [14]. In addition, the available anti-CGRP Mabs are not interchangeable with respect to administration route, dosing frequency, and onset profile, characteristics that may meaningfully influence both patient preference and real-world effectiveness [15]. For example, intravenous eptinezumab may be preferentially selected for patients in whom a rapid onset of effect is clinically desirable or for those with adherence concerns, whereas monthly subcutaneous agents may be better suited to patients who prioritize home administration and dosing autonomy [16].

Despite the increasing use of CGRP-targeted therapies, comparative evidence against established oral preventives remains largely derived from indirect comparisons and network meta-analyses, which necessarily pool heterogeneous trial populations, endpoints, and follow-up durations [7,17]. While informative at a population level, these analyses cannot fully substitute for direct, head-to-head comparisons, particularly when the comparator is associated with a distinct tolerability and discontinuation profile [18]. Pragmatic, real-world comparative studies are therefore of particular clinical value, as they integrate efficacy, safety, and treatment persistence under routine conditions, reflecting long term outcomes. This approach is especially relevant in tertiary headache centers, where referral bias toward more severe and treatment-refractory migraine is expected, and where differences in adverse-event burden and discontinuation rates may substantially influence disability and quality-of-life (QOL) outcomes [19].

As such, using retrospective data from Greek specialized headache clinics within the Greek Research Alliance for the Study of headache and Pain (GRASP) network, we evaluated outcomes in adult patients with episodic migraine (EM) or CM treated with either topiramate or one of the commercially available anti-CGRP Mabs. The primary aim was to provide a pragmatic intention-to-treat assessment of comparative effectiveness between anti-CGRP Mabs versus topiramate for migraine prophylaxis over a six-month treatment period, with particular attention to treatment discontinuation. An additional primary objective was to describe outcomes across individual anti-CGRP Mabs to elucidate potential within-class differences in terms of efficacy and safety.

## 2. Materials and Methods

On an intention-to-treat basis, this retrospective, multicenter, real-world study included adult Greek patients with either EM or CM who received either topiramate or one of the four anti-CGRP Mabs for a treatment duration of six months. Treatment selection was based on the treating physician’s clinical judgment and patient preferences.

The diagnosis of migraine was established in accordance with the 2018 criteria of the International Classification of Headache Disorders, third edition [20]. Participants were selected, treated, and clinically followed by headache specialists within the GRASP network across major urban geographical regions of Greece (9 cities, 12 recruitment sites).

The administered preventive medications included: (i) fremanezumab 225 mg subcutaneously (SC) monthly; (ii) erenumab 140 mg SC monthly; (iii) galcanezumab 240 mg SC as a loading dose followed by five monthly maintenance doses at 120 mg SC; and (iv) eptinezumab 100 mg administered intravenously on a quarterly basis. Patients at 300 mg eptinezumab were not included, because in our clinical practice this dose increment is considered only in partial responders after two quarterly given sessions with 100 mg eptinezumab. Patients treated with topiramate received individualized dosing according to standard clinical practice, as first or second-line treatment, with gradual titration to the lowest effective dose or until a maximum daily dose of 200 mg was reached, or until treatment discontinuation due to adverse events. The median topiramate daily dosage was 100 mg (range: 75–200 mg).

In order to be included in this study, patients with a confirmed diagnosis of EM or CM, with or without aura or MOH, were required to have received treatment with either the above-mentioned anti-CGRP Mabs or topiramate, in line with the approved indications and national reimbursement policies. Eligibility for anti-CGRP Mabs therapy required failure, defined as an inadequate response to, or intolerance of, or contraindication to, at least three prior preventive treatments. As, during the period covered in this study, flunarizine, one of the three required preventives needed to have failed was no longer commercially available in Greece, two prior failures were then required for reimbursement. Topiramate was permitted as either a first- or second-line preventive treatment.

As part of routine clinical practice, patients were asked to maintain a daily paper-based headache diary during the baseline period (3 months prior to the onset of each intervention; T0) and throughout follow-up visits at 3 months (T1) and 6 months (T2) after treatment initiation. To be considered compliant, patients were required to complete the diary for at least 75% of the total 180-day observation period. Headache diaries were used to capture changes in monthly headache days (MHD), the number of MHD with peak pain intensity ≥5 on a 0–10 visual analog scale (VAS) [21], and the monthly intake of abortive medications (MAI) for acute migraine attacks. Migraine-related disability was assessed longitudinally from baseline to month 6 using the Migraine Disability Assessment (MIDAS) and the Headache Impact Test-6 (HIT-6) [22,23,24]. Health-related quality of life (QOL) was evaluated using the EQ-5D visual analog scale (EQ-VAS), a thermometer-like vertical scale on which patients self-rated their perceived health status, ranging from 0–40 (severely impaired) and 50–70 (moderately impaired) to 100 (best imaginable health status) [25]. Based on a review of patients’ medical records, treatment efficacy and disability outcomes at month 6 were calculated in comparison to baseline using data derived from the final month of treatment.

Patients were instructed to report any adverse events (AEs) either spontaneously or in response to structured questioning during telephone follow-up conducted between days 0 and 21 after administration of each anti-CGRP or every 40 days during topiramate treatment. All local and systemic AEs, as well as any serious adverse events, were documented in the patients’ medical records and subsequently evaluated by the treating physician for potential causal association with each intervention. Rates of treatment discontinuation due to an adverse event (DAE) before month-6, were also recorded.

The primary outcome was the reduction in MHDs over the 6-month treatment period for each intervention, with the aim of providing comparative evidence of efficacy between anti-CGRP Mabs and topiramate. The co-primary outcome was the comparison of responder rates, defined as the proportion of patients achieving a ≥50% reduction in MHDs (50% response) and a ≥75% reduction in MHDs (75% super-response), for each anti-CGRP Mab compared to topiramate. Secondary outcomes included: (i) changes in migraine-related disability and QOL metrics from baseline to month 6, assessed using MIDAS, HIT-6, and EQ-VAS; (ii) changes in the number of MHDs with peak headache intensity ≥5 on VAS; (iii) changes in MAI; and (iv) differences in treatment discontinuation rates due to safety or tolerability issues.

The study was conducted in accordance with the Declaration of Helsinki. The study protocol was approved by the Institutional Review Board of General Clinic, Thessaloniki (Project ID: 8190—CLO00919a, 26 November 2025). An opt-out consent procedure was permitted from the Institutional Review Board of “General Clinic, Thessaloniki”, owing to the retrospective, file-based nature of the study. All data were collected and analyzed in anonymized form, with no personally identifiable information recorded. Data handling and storage complied with national regulations and the General Data Protection Regulation. This retrospective study was not registered in a public clinical trial registry, as it constitutes part of the GRASP study group’s ongoing migraine registry.

As described above, the study applied an intention-to-treat design, ensuring that all participants were included in the final analysis based on their initial treatment assignment. This approach was maintained even if participants withdrew prematurely for any reason, including safety concerns or issues related to treatment tolerability. Nonetheless, the research protocol required that each intervention be administered for a minimum of six months before efficacy was evaluated. Reasons for treatment discontinuation prior to the six-month efficacy assessment were systematically recorded from patients’ medical files and those who were lost to follow-up for any reason were classified as non-responders per se.

### Statistical Analysis

Using descriptive statistics, categorical variables are presented as observed counts and weighted percentages, while continuous variables are reported as either mean or median with the corresponding standard error or range, as appropriate. The normality of the data was assessed using the Kolmogorov–Smirnov and Shapiro–Wilk tests. Comparisons between groups were performed using the Mann-Whitney U test, while the effect sizes were calculated using rank-biserial correlations. For within-group comparisons (baseline vs. month 6 in the same patients), the Wilcoxon signed-rank test for paired data was used. Continuous variables with a non-normal distribution are presented as median and interquartile range (IQR). A subgroup analysis was performed to compare ≥50% responder rates (reduction in MHDs) between patients with CM and those with HFEM, defined as baseline MHDs >8. The same analysis was performed between patients with MOH and those without MOH. Responder status was treated as a binary variable. Group comparisons were conducted using the chi-square test or Fisher’s exact test, where appropriate. Unless otherwise stated, all tests were two-sided, and significance was set at *p* < 0.05. SPSS for Windows (release 29.0; SPSS Inc., Chicago, IL, USA) conducted the statistics.

## 3. Results

### 3.1. Demographic and Clinical Migraine Characteristics of Participants

The group consisted of 409 adult migraine patients, with a median age of 45.2 years (range 20–78), and predominantly comprised females (80%). The median duration of migraine was 21.6 years, reflecting a long-standing disease burden, and 12.2% of patients reported migraine with aura. Most participants were diagnosed with EM (68%), while nearly one-third (32%) had CM. At baseline, patients with both EM and CM experienced a substantial migraine-related burden, with a pooled median of 14.9 MHDs and a median MAI of 13.3. The median of moderate/severe MHDs (VAS ≥ 5/10) was 11. Disability and impact scores were high, as shown by a median MIDAS score of 73 and a median HIT-6 score of 67.2 to indicate severe migraine-related disability and impact on daily functioning. MOH was evident in 38.6% of participants.

Comorbidities were common, particularly psychiatric, including anxiety (*n* = 108; 26.4%), depression (*n* = 52; 12.7%), bipolar disorder (*n* = 5; 1.2%), and combined anxiety and depression (*n* = 77; 18.8%). MOH was present in 158 patients (38.6%). Finally, more than half of the patients had previously tried one to three preventive treatments, while 35.5% had been exposed to more than four, highlighting a largely treatment-experienced population.

Regarding treatment allocation, patients had received either topiramate (27.4%) or one of the anti-CGRP Mabs, including fremanezumab (22.5%), erenumab (14.4%), galcanezumab (20.0%), and eptinezumab (15.6%). The baseline migraine phenotypes across interventions were broadly similar, with corresponding mean ages ranging from 42 to 47 years. At baseline, the median MHDs were comparable among anti-CGRP Mabs groups (15.2) and topiramate (14.2). MIDAS scores indicated severe disability, overall, appearing higher in the anti-CGRP Mabs groups, compared to topiramate (sum median score of 79 vs. 59, respectively). Table 1 summarizes the baseline demographic and clinical characteristics of the study population across treatment arms.

### 3.2. Efficacy Analysis

Within the six months treatment period, five patients in the erenumab group and 32 in the topiramate group had discontinued their treatment because of adverse events/intolerability and were hence considered as non-responders. The reasons for all drop-outs are described below.

A reduction in mean MHDs was observed across all interventions in the 6-month assessment period. Baseline mean MHDs were 14.9 and at 6 months post-treatment were 8.2 (−6.7 days) for all types of prophylactic treatments combined. The mean MHDs for the anti-CGRP Mabs group (*n* = 297) were 15.2 at baseline and 7.4 at 6 months post treatment (mean reduction: −7.8; median reduction: −8). For the topiramate group they were 14.2 at baseline (*n* = 112) and 10.4 (mean reduction: −3.8; median reduction: −4), at 6 months post-treatment.

All four anti-CGRP Mabs demonstrated large effect sizes for monthly headache day reduction, ranging from −0.81 to −0.85. These effect sizes were consistently greater than those observed with topiramate, which showed a more modest effect size of −0.64. All within-treatment comparisons versus baseline were highly statistically significant, with *p* values < 0.001 (Wilcoxon signed-rank test). Direct comparisons with topiramate using the Mann–Whitney U test showed strongly mean negative z values (z = −4.208) for all anti-CGRP Mabs, with corresponding *p* values < 0.001, to document the superiority of these biological agents over a traditional oral preventive in our cohort. The median MHD reductions were comparable across anti-CGRP Mabs with median drops of −7.5, compared to the much lower reduction seen with topiramate, i.e., −3. Response (>50% MHD reduction) and super-response (>75% MHD reduction) rates were markedly higher with the use of each anti-CGRP Mab than those obtained with topiramate. Table 2 summarizes the comparative effectiveness of anti-CGRP Mabs in reducing MHDs and improving responder rates, with topiramate used as the reference comparator.

**Figure 1:**
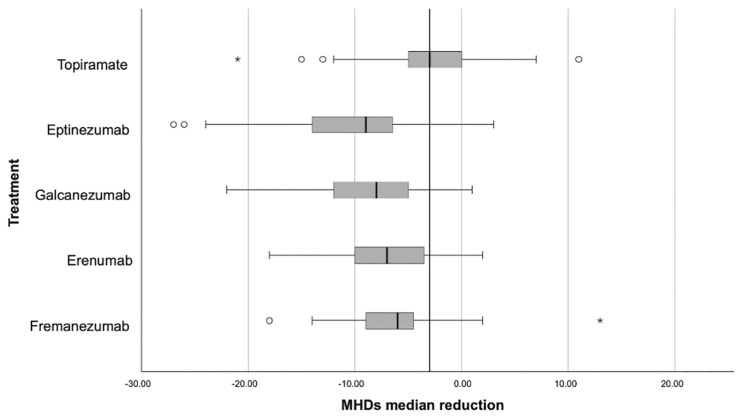
Box plot showing that the median MHDs reduction was greater with each anti-CGRP Mab than with topiramate (*p* < 0.001, Mann–Whitney U test). Open circles indicate outliers (1.5–3 × IQR), and asterisks indicate extreme values (>3 × IQR).

As shown in Figure 1, each anti-CGRP Mab achieved a greater median reduction in monthly headache days than topiramate, with all comparisons reaching statistical significance (Mann–Whitney; *p* < 0.001). The four anti-CGRP Mabs targeting the CGRP pathway showed closely aligned median reductions, clustering around a moderate/high decrease in MHDs after six months of treatment. In contrast, topiramate showed a much wider range of responses.

While some topiramate-treated patients experienced marked improvements, others benefited far less, reflecting what is commonly seen in clinical practice, where side effects and tolerability often influence how well patients respond to topiramate.

The ≥50% responder rate, relating to MHD reduction, was numerically lower in patients with CM, compared to those with HFEM (56.4% vs. 63.6%), although this difference did not reach statistical significance (*p* = 0.280). In contrast, patients with MOH demonstrated significantly lower ≥50% responder rates, compared to those without MOH (53.1% vs. 67.1%, *p* = 0.021).

Regarding MAI reduction, all CGRP-targeting Mabs demonstrated moderate to large effects. Galcanezumab showed the largest reduction with an effect size of −0.85, followed by Erenumab at −0.79 and Eptinezumab at −0.73. Fremanezumab also achieved a clinically meaningful reduction, though of slightly smaller magnitude at −0.64. In comparison, topiramate showed a more modest effect size of −0.60.

For MHDs with peak severity of ≥5 in VAS pain, the strongest effect was observed with fremanezumab and galcanezumab, which both showed large effect sizes of −0.83 and −0.85, respectively, indicating a pronounced reduction in the most disabling migraine attacks. Erenumab also demonstrated a substantial effect at −0.78, while eptinezumab shows a moderate-to-large effect of −0.68. Topiramate demonstrated a smaller, though still statistically significant, effect of −0.62. Table 3 demonstrates the comparative effect sizes and statistical significance of anti-CGRP Mabs and topiramate on MAI as well as on moderate-to-severe MHDs with VAS pain of ≥5/10.

Furthermore, Table 4 describes the effect sizes obtained from each treatment in reducing the migraine-related disability and QOL outcomes, assessed by MIDAS, HIT-6, and EQ-VAS. Across all outcomes, negative effect sizes indicated improvement from baseline, and the associated *p* values confirm that these changes were statistically significant.

Specifically for disability, assessed with the MIDAS score, all treatments were associated with significant reductions. The CGRP-targeting Mabs showed large effect sizes, ranging from −0.76 to −0.84, consistent with substantial reductions in migraine-related disability. In contrast, topiramate demonstrated a more moderate effect size of −0.60, indicating improvement but of smaller magnitude. All comparisons reach high statistical significance, with *p* values of at least < 0.002.

A similar pattern was observed for the HIT-6 scores, where the anti-CGRP Mabs again obtained large effect sizes, particularly eptinezumab at −0.84 and galcanezumab at −0.78, suggesting marked improvements in perceived headache impact. Fremanezumab and erenumab also showed clinically meaningful effects, albeit slightly smaller. Topiramate showed a modest reduction with an effect size of −0.50, reinforcing the impression that CGRP-targeted therapies yield greater benefits on patient-perceived impact than the traditional oral preventive.

The QOL status, measured using the EQ-VAS, revealed greater differentiation between treatments. Galcanezumab and eptinezumab demonstrated marked improvements, post-treatment, with effect sizes of −0.77 and −0.84, respectively, indicating a pronounced effect on patient’s QOL status at 6 months post-intervention. Erenumab showed a moderate effect size of −0.64, while fremanezumab exhibited a slightly smaller, but still statistically significant effect, of −0.58. Topiramate again showed a poorer reduction effect size of −0.22 in terms of QOL improvement. Despite these differences in magnitude, all treatments achieved statistically significant reductions, underscoring a consistent beneficial effect.

### 3.3. Safety Analysis

Overall, the interventions demonstrated a favorable safety profile, with low rates of SAEs during the first six months, except for topiramate, which was associated with substantially higher rates of both AEs and DAEs. In total, 37 AEs led to treatment discontinuation, including five patients in the erenumab group and 32 in the topiramate group. Discontinuations in the erenumab group were exclusively due to de novo constipation, whereas those in the topiramate group were primarily attributed to distal paresthesia, weight loss, cognitive impairment, and urolithiasis.

The proportion of patients reporting any AE was 13% with fremanezumab (*n* = 12), 23.7% with erenumab (*n* = 14), 20.7% with galcanezumab (*n* = 17), 14.1% with eptinezumab (*n* = 9), and 58% with topiramate (*n* = 65). The most frequently reported AEs were cognitive dysfunction (*n* = 23) and distal numbness (*n* = 25), both occurring predominantly in the topiramate group. Injection-site pain or swelling was reported in 23 anti-CGRP Mabs-treated patients overall, mainly among those receiving galcanezumab (*n* = 19), with fewer cases observed in the fremanezumab (*n* = 2), erenumab (*n* = 2), and eptinezumab (*n* = 3) groups. However, there were no treatment discontinuations among patients treated with subcutaneous GCRP-targeted therapies, because of injection-site pain or swelling.

## 4. Discussion

Real-world evidence can address several clinically important unanswered questions in the setting of migraine prophylaxis. First, it can clarify the extent to which anti-CGRP Mabs outperform topiramate in terms of overall effectiveness and safety over a clinically meaningful timeframe of six months, when titration, adherence, and treatment persistence have stabilized. Second, it can explore whether individual CGRP-targeted Mabs demonstrate differential outcomes in routine practice, when prescribed according to physician judgment and patient preference.

In this large, multicenter, real-world retrospective study conducted within the GRASP network, anti-CGRP Mabs demonstrated superior effectiveness and tolerability compared with topiramate in adult patients with migraine. Our cohort represents a highly treatment-experienced population with long-standing disease, substantial baseline headache burden, high disability, a high prevalence of MOH and psychiatric comorbidities, reflecting the complexity routinely encountered in specialized headache clinics.

Across six months of treatment, all preventive strategies were associated with significant reductions in MHDs; however, the magnitude of benefit was consistently greater with anti-CGRP Mabs than with topiramate. The mean reduction of nearly eight MHDs observed with anti-CGRP therapies is clinically meaningful and aligns with, or exceeds, reductions reported in randomized controlled trials and other real-world cohorts of refractory migraine populations. In contrast, the more modest reduction observed with topiramate mirrors real-life experience, where efficacy is often limited by tolerability and adherence rather than pharmacological inefficacy alone. Direct comparisons with topiramate showed quite significant effect sizes and corresponding *p* values favoring each CGRP-targeted Mab. This confirms that the distributions of MHD reductions for each anti-CGRP Mabs are significantly shifted toward greater benefit compared with topiramate, reinforcing the superiority of these agents over a traditional oral preventive in our cohort. Another key finding is the relative consistency of all CGRP-targeted Mabs, i.e., eptinezumab, galcanezumab, erenumab, and fremanezumab. Their interquartile ranges are comparatively narrow and heavily overlapping, indicating broadly comparable effectiveness in terms of response, thoroughly supporting the view that no single anti-CGRP Mab clearly outperforms the others with respect to MHD reduction. However, these differences seem too insignificant to be translated into meaningful clinical distinctions for most patients. Hence, this clustering suggests a class effect, where targeting the CGRP pathway yields predictable and consistent improvements across migraine patients.

Responder and super-responder analyses further reinforce the superiority of CGRP-targeted therapies. Significantly higher proportions of patients achieved ≥50% and ≥75% reductions in MHDs with each anti-CGRP Mab compared with topiramate. Importantly, the four anti-CGRP agents demonstrated closely aligned effect sizes and overlapping interquartile ranges, again suggesting a class effect rather than clinically meaningful differences between individual molecules. This finding supports current clinical practice, where selection among anti-CGRP Mabs is often guided by patient preference, route of administration, comorbidities, and reimbursement considerations rather than expectations of differential efficacy. Nonetheless, while all four anti-CGRP agents showed meaningful reductions in MHDs and comparable responder rates, caution is needed in robustly supporting statistical equivalence, because of the observed variability in IQRs, with fremanezumab’s IQR of 4.75 and eptinezumab’s of 7.75.

Beyond headache frequency, anti-CGRP Mabs consistently outperformed topiramate in reducing moderate-to-severe headache days and MAIs, outcomes that are particularly relevant in patients with MOH. The observed reductions in MAI suggest that effective CGRP-targeted prevention may facilitate withdrawal from excessive acute medication use; a finding that is clinically relevant given the risks of medication overuse and its contribution to the chronification of migraine [26].

Furthermore, disability and patient-reported outcomes showed marked improvement with anti-CGRP therapies. Large effect sizes were observed across MIDAS and HIT-6 scores, indicating substantial reductions in migraine-related disability and perceived headache impact. Improvements in QOL, as measured by EQ-VAS, were also more pronounced with anti-CGRP Mabs, particularly galcanezumab and eptinezumab. In contrast, topiramate demonstrated only modest effects on disability and minimal improvement in QOL, underscoring the disconnect that can exist between headache frequency reduction and broader patient-centered outcomes when tolerability is suboptimal.

Safety and tolerability emerged as key differentiators between treatment strategies. Among anti-CGRP therapies, we found a significant difference in injection-site reactions between galcanezumab and other subcutaneous ligand-targeting CGRP agents, such as fremanezumab. Although no relevant treatment discontinuations occurred, this finding could be an important consideration for patient selection, especially when choosing between different anti-CGRP agents. While anti-CGRP Mabs were, otherwise, generally well tolerated, topiramate was associated with substantially higher rates of adverse events and treatment discontinuation. Cognitive dysfunction, distal paresthesia, weight loss, and urolithiasis were prominent contributors to topiramate discontinuation, consistent with its known adverse event profile. Although erenumab showed a higher rate of adverse event-related discontinuation compared with other anti-CGRP agents, primarily due to constipation, overall discontinuation rates for CGRP-targeted therapies remained low. These findings highlight the clinical importance of tolerability in sustaining long-term preventive treatment and achieving meaningful outcomes in real-world practice.

In agreement with our findings, overall, and the most directly comparable previously published evidence is the HER-MES trial, a 24-week randomized, double-blind, double-dummy head-to-head comparison of erenumab versus topiramate for migraine prevention. It demonstrated substantially lower discontinuation due to AEs with erenumab (10.6%) versus topiramate (38.9%) and higher ≥50% responder rates with erenumab (55.4% vs. 31.2%) [27]. A later post hoc analysis focused on patient-reported outcomes at week 24 (HIT-6, SF-36) also favored erenumab over topiramate [28]. As such, the strengths of our real-world study include its large sample size, multicenter design, standardized outcome assessment using validated instruments, and inclusion of a comparator oral preventive frequently used in routine care, contrary to the vast majority of comparative literature that is dominated by indirect comparisons and network meta-analyses, including topiramate vs. CGRP-targeted Mabs [7,29,30,31], but are not real-world head-to-head cohorts. Methodology papers in this area also underscore that much of the comparative inference relies on indirect treatment comparison methods (including matching-adjusted indirect comparisons and other approaches) to bridge differences in trial design and mode of administration [32], which further highlights the paucity of pure real-world head-to-head cohort evidence.

Nevertheless, several limitations must be acknowledged. The retrospective, non-randomized design introduces potential selection bias and confounding by indication, particularly given that anti-CGRP Mabs were reserved for patients with multiple prior preventive failures. Headache diary data were paper-based, which may introduce reporting bias, and follow-up was limited to six months, precluding assessment of long-term sustainability of benefits. Another limitation concerns that anti-CGRP Mabs were restricted to patients who had failed at least three prior preventive treatments, while topiramate was permitted as a first or second-line treatment; thus imposing a selection bias to challenge the generalizability of the results. Furthermore, the absence of adjustment for baseline differences in disability and treatment history may have influenced comparative estimates, although the consistency of findings across multiple outcomes strengthens the validity of our findings. The lack of multiple testing corrections might also be perceived as a limitation, although the minor differences observed are unlikely to meaningfully impact the overall efficacy or safety profile of treatments.

We performed an intention-to-treat analysis to provide a pragmatic estimate of each treatment effect in real-world practice, where perfect adherence is often not achieved. Tellingly, attrition bias stands as the most important consideration in the interpretation of our findings, given the differential discontinuation rates observed between treatment groups. Although all interventions were intended to be administered for a minimum of six months, early treatment discontinuation occurred much more frequently with topiramate (32/112; 28.6%) and, to a lesser extent, with erenumab (5/59; 8.5%), primarily due to adverse events and poor tolerability. In this study, patients who discontinued treatment early or were lost to follow-up were conservatively classified as non-responders. Based on prior evidence [27,28], coupled with our routine clinical experience, higher discontinuation rates due to AEs were anticipated with topiramate. For this reason, we deliberately included a larger topiramate-treated cohort (*n* = 112) than the largest anti-CGRP Mab group, i.e., fremanezumab (*n* = 92). Tellingly, this approach likely mitigates, but does not fully eliminate, attrition bias as the higher discontinuation rate in the topiramate group may have led to an underestimation of its potential efficacy among patients who could tolerate and persist with therapy, while simultaneously reflecting real-world clinical effectiveness, where adherence is a significant determinant of outcome. Conversely, the lower attrition observed with most anti-CGRP Mabs may have favored more complete outcome data in these groups, potentially amplifying observed between-group differences. Importantly, however, attrition itself represents a clinically meaningful outcome in migraine prevention, as tolerability and treatment persistence directly influence long-term effectiveness. The substantial difference in discontinuation rates therefore supports, rather than undermines, the external validity of the findings. However, the possibility that differential attrition contributed to effect size inflation cannot be excluded, and the results should be interpreted in light of this limitation. Nonetheless, and despite these limitations, our study provides valuable real-world evidence regarding the comparative effectiveness and tolerability of the most used preventative treatments for migraine in a complex patient population with substantial treatment histories.

## 5. Conclusions

This real-world, retrospective study provides evidence that anti-CGRP Mabs offer superior effectiveness and greater improvements in disability and QOL as well as better tolerability, compared head-to-head with topiramate in migraine patients. Our findings make anti-CGRP Mabs particularly attractive for patients who have failed or poorly tolerated traditional oral preventives, while topiramate remains a viable option, especially given cost and accessibility considerations, but requires careful patient selection and monitoring. Finally, the broadly comparable performance of individual anti-CGRP agents generally supports a class-wide benefit in terms of consistency though not equivalence, reinforcing their role as a preferred preventive option in treatment-resistant migraine populations encountered in specialist headache care. However, considering the observed variability in IQRs across anti-CGRPs, the latter assumption should be cautiously interpreted before larger, head-to-head studies with pre-defined handling of missing data can indeed demonstrate whether these agents are interchangeable in terms of efficacy in real-world practice.

## Figures and Tables

**Table 1:** Basic demographic and clinical characteristics of our cohort, per treatment arm.

	Pooled (*n*) (*n* = 409)	Fremanezumab (*n* = 92)	Erenumab (*n* = 59)	Galcanezumab (*n* = 82)	Eptinezumab (*n* = 64)	Topiramate (*n* = 112)
Variable						
Age	45.2 (20–78)	45.5 (22–66)	46 (25–65)	47 (26–70)	46.6 (21–78)	42.4 (20–70)
Female (*n*, %)	329 (80)	72 (78.3)	43 (72.9)	70 (85.4)	54 (84.4)	90 (80.4)
Migraine type						
Episodic (*n*, %)	278 (68)	82 (89)	34 (57.6)	51 (62.2)	36 (56.3)	75 (67)
Chronic (*n*, %)	131 (32)	10 (11)	25 (42.4)	31 (37.8)	28 (43.8)	37 (33)
Baseline MHD						
Mean (range)	14.9 (5–30)	12.5 (5–24)	16.6 (7–30)	15.8 (8–30)	16.9 (8–30)	14.2 (5–30)
Baseline MIDAS						
Median (range)	73 (6–260)	70.7 (12–199)	76.7 (10–260)	83.6 (25–170)	81.8 (19–160)	59 (6–198)
MOH (*n*, %)	158 (38.6)	29 (31.5)	25 (42.4)	40 (48.8)	36 (56.3)	46 (41.1)
Number of previously used treatments						
none	46 (11.2)	0	0	0	0	46
1–3	217 (53.1)	56	27	41	31	62
>4	146 (35.7)	35	32	38	33	8

**Table 2:** Comparative effectiveness of anti-CGRP Mabs for reducing MHD and responder rates, using topiramate as the reference.

	Effectiveness in Reducing MHDs				Comparison with Topiramate		
Treatment	Effect Size	*p*-Value ^a^	Median Reduction	IQR	z ^b^	*p*-Value	Response ^c^/Super-Response Rate ^d^
Fremanezumab	−0.81	<0.001	−6	4.75	−5.965	<0.001	72.8%/21.7%
Erenumab	−0.82	<0.001	−7	5.25	−5.31	<0.001	55.9%/22%
Galcanezumab	−0.85	<0.001	−8	7	−7.448	<0.001	65.9%/22%
Eptinezumab	−0.85	<0.001	−9	7.75	−7.617	<0.001	81.3%/29.7%
Topiramate	−0.64	<0.001	−3	5			30.4%/6.3%

^(a)^ Rank-biserial correlations; ^(b)^ Mann–Whitney U; ^(c)^ greater than 50% reduction in MHDs; ^(d)^ greater than 75% reduction in MHDs.

**Table 3:** Effect sizes and statistical significance of preventive treatments on monthly acute intake (MAI) and moderate-to-severe MHDs with VAS ≥ 5/10.

Intervention	MAI		Moderate/Severe MHDs (VAS ≥ 5/10)	
	Effect Size	*p*-Value	Effect Size	*p*-Value
Fremanezumab	−0.64	<0.001	−0.83	<0.001
Erenumab	−0.79	<0.001	−0.78	<0.001
Galcanezumab	−0.85	<0.001	−0.85	<0.001
Eptinezumab	−0.73	<0.001	−0.68	<0.001
Topiramate	−0.6	<0.001	−0.62	<0.001

**Table 4:** Effect sizes and statistical significance of interventions on disability, headache impact, and QOL outcomes.

Intervention	MIDAS Reduction		HIT-6 Reduction		EQ-VAS Improvement	
	Effect Size	*p*-Value	Effect Size	*p*-Value	Effect Size	*p*-Value
Fremanezumab	−0.83	<0.001	−0.75	<0.001	−0.58	0.008
Erenumab	−0.76	0.002	−0.66	<0.001	−0.64	<0.001
Galcanezumab	−0.84	<0.001	−0.78	<0.001	−0.77	<0.001
Eptinezumab	−0.83	<0.001	−0.84	<0.001	−0.84	<0.001
Topiramate	−0.6	<0.001	−0.5	<0.001	−0.22	0.002

## Data Availability

The original contributions presented in this study are included in the article. Further inquiries can be directed to the corresponding author.

## Abbreviations

The following abbreviations are used in this manuscript:

HFEM	High-frequency episodic migraine
CM	Chronic migraine
MOH	Medication-overuse headache
anti-CGRP Mabs	Monoclonal antibodies targeting the calcitonin gene-related peptide
QOL	Quality-of-life
GRASP	Greek specialized headache clinics within the Greek Research Alliance for the Study of headache and Pain
EM	Episodic migraine
MHD	Monthly headache days
VAS	MHD with peak pain intensity ≥5 on a 0–10 visual analog scale
MAI	Monthly intake of abortive medications
MIDAS	Migraine Disability Assessment
HIT-6	Headache Impact Test-6
EQ-VAS	EQ-5D visual analog scale
AEs	Adverse events
DAEs	Discontinuation due to an adverse event
IQR	Interquartile range
